# Submarine Groundwater Discharge helps making nearshore waters heterotrophic

**DOI:** 10.1038/s41598-018-30056-x

**Published:** 2018-08-03

**Authors:** Shu-Lun Wang, Chen-Tung Arthur Chen, Ting-Hsuan Huang, Hsiao-Chun Tseng, Hon-Kit Lui, Tsung-Ren Peng, Selvaraj Kandasamy, Jing Zhang, Liyang Yang, Xuelu Gao, Jiann-Yuh Lou, Fu-Wen Kuo, Xue-Gang Chen, Ying Ye, Yi-Jie Lin

**Affiliations:** 1 Department of Marine Environmental Engineering, National Kaohsiung University of Science and Technology, Nanzih Campus., Kaohsiung, 811 Taiwan; 2 Department of Oceanography, National Sun Yat-sen University, Kaohsiung, 804 Taiwan; 3 UNESCO UNITWIN/WiCop. Physical Chemistry Department. Faculty of Marine and Environmental Sciences, Polígono río San Pedro s/n, University of Cadiz, 11519 Puerto Real, Cadiz Spain; 4 Taiwan Ocean Research Institute, National Applied Research Laboratories, Kaohsiung, 801 Taiwan; 5 Department of Soil and Environmental Sciences, National Chung Hsing University, Taichung, 402 Taiwan; 6 State Key Laboratory of Marine Environmental Science, Xiamen University, Xiamen, 361102 Fujian, China; 7 Graduate School of Science and Engineering, University of Toyama, 3190 Gofuku, Toyama, 930-8555 Japan; 8 College of Environment and Resources, Fuzhou University, Fuzhou, 350116 Fujian, China; 9 CAS Key Laboratory of Coastal Zone Environmental Processes and Ecological Remediation, Yantai Institute of Coastal Zone Research, Chinese Academy of Sciences, Yantai, 264003 Shandong China; 10 Department of Marine Science, Republic of China Naval Academy, Kaohsiung, 813 Taiwan; 11 National Museum of Marine Biology & Aquarium, Pingtung, 944 Taiwan; 12 Ocean College, Zhejiang University, Hangzhou, 310058 Zhejiang, China

## Abstract

Submarine groundwater discharge (SGD) is the submarine seepage of all fluids from coastal sediments into the overlying coastal seas. It has been well documented that the SGD may contribute a great deal of allochthonous nutrients to the coastlines. It is, however, less known how much carbon enters the ocean via the SGD. Nutrients (NO_3_, NO_2_, NH_4_, PO_4_, SiO_2_), alkalinity and dissolved inorganic carbon (DIC) in the submarine groundwater were measured at 20 locations around Taiwan for the first time. The total N/P/Si yields from the SGD in Taiwan are respectively 3.28 ± 2.3 × 10^4^, 2.6 ± 1.8 × 10^2^ and 1.89 ± 1.33 × 10^4^ mol/km^2^/a, compared with 9.5 ± 6.7 × 10^5^ mol/km^2^/a for alkalinity and 8.8 ± 6.2 × 10^5^ mol/km^2^/a for DIC. To compare with literature data, yields for the major estuary across the Taiwan Strait (Jiulong River) are comparable except for P which is extremely low. Primary production supported by these nutrient outflows is insufficient to compensate the DIC supplied by the SGD. As a result, the SGD helps making the coastal waters in Taiwan and Jiulong River heterotrophic.

## Introduction

Submarine groundwater discharge (SGD) is the submarine seepage of all fluids from coastal sediments into the overlying coastal areas. It has been well documented that the SGD may contribute much nutrients to the coastlines^1–9^. Excessive supply of nutrients may lead to eutrophication, hence affecting the sustainability of the coastal environment^10^. SGD also contains excess carbon^11–14^. It is, however, less known how nutrients and carbon interact after the SGD enters the oceans.

Because the groundwater has been in contact with the sediments for a long period of time it is expected that some of the particulate organic matter in the sediments would have decomposed thus consuming dissolved oxygen (DO) but releasing dissolved inorganic carbon (DIC) and dissolved organic carbon (DOC) along with nutrients. The partial pressure of CO_2_ (pCO_2_) would also increase. Part of the DOC would decompose, further increase DIC and pCO_2_. Some of the CaCO_3_ in the sediments might also dissolve thus increase the total alkalinity (TA). The groundwater is isolated from the atmosphere but when the groundwater enters the oceans it is expected that the high pCO_2_ in the SGD might make the receiving coastal water a CO_2_ source for the atmosphere. Yet, the nutrient supply from the SGD would enhance primary productivity in coastal waters, hence drawing down the pCO_2_ of surface waters. Whether the SGD would eventually lead to a carbon source or sink into the receiving coastal waters does not have an a priori answer.

Recently the salinity and major ions such as Ca, Mg, K, Na, Cl and SO_4_ in the submarine groundwater samples around Taiwan have been measured^15^. However, nutrients and carbon in the SGD have never been reported in the SGD from this part of the world. In fact, only a handful of studies have reported nutrients and carbon in the SGD in China^7,16–19^. In this study, SGD samples were collected from 20 locations around the subtropical island with an area of 35,873 km^2^. DO, nutrients (NO_3_, NO_2_, NH_4_, PO_4_, SiO_2_), N_2_O, CH_4_, DOC, pH, TA, and DIC were measured and pCO_2_ calculated. For comparison, we also collected data in the Jiulong River in China across the Taiwan Strait. Whether the SGD helps making the coastal waters autotrophic or heterotrophic is evaluated.

## Results and Discussion

### Concentrations of chemical parameters

**Figure 1:**
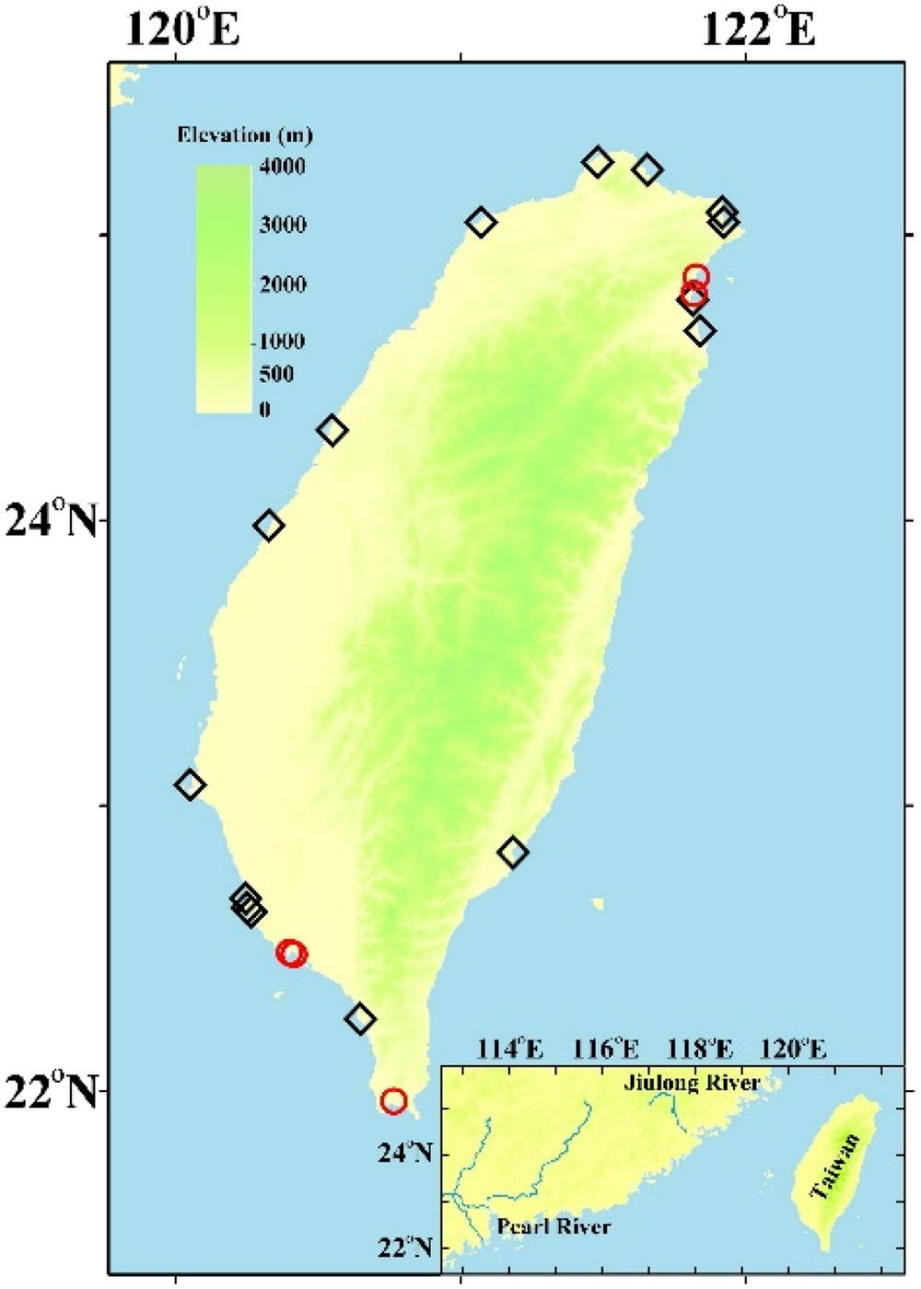
Sampling locations.

**Figure 2:**
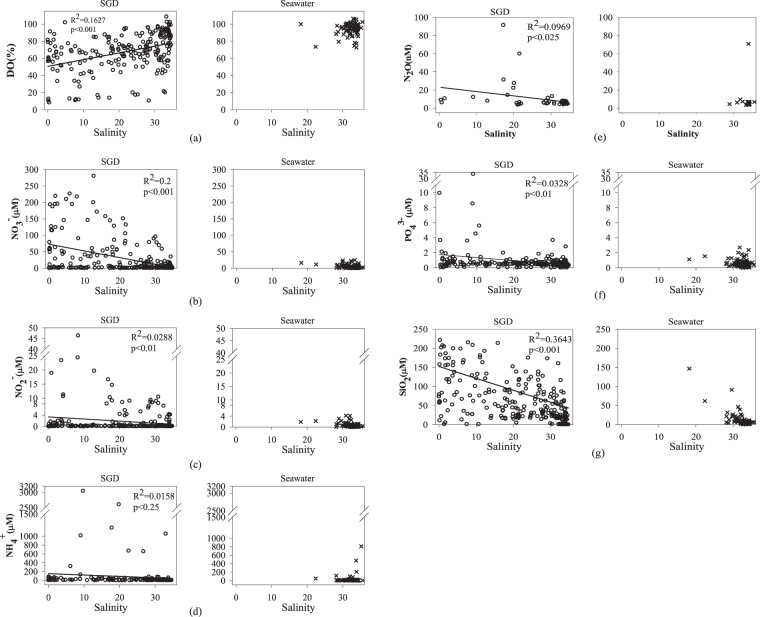
(**a**) Percentage DO saturation, (**b**) NO_3_, (**c**) NO_2_, (**d**) NH_4_, (**e**) N_2_O, (**f**) PO_4_, and (**g**) SiO_2_, vs salinity in the submarine groundwater and local surface seawater, respectively.

The average concentrations of chemical parameters for a total of 278 submarine groundwater measurements and for the local surface seawaters above the SGD sampling sites are given in Table 1. Salinity data are taken from Chen *et al*.^15^. Out of the 20 sampling sites 15 showed evidence of some submarine freshwater outflow (those marked is black on Fig. 1). The salinity of the SGD varies widely between 0.008 and 34.8 with an average of 21.92 ± 11.43, which is considerably lower than the average of the corresponding local surface seawater (32.5 ± 2.42). Previous study^15^ has indicated that the SGD export from Taiwan is about 1.07 ± 0.7 × 10^10^ t/a; of which, 0.38 ± 0.48 × 10^10^ t/a is the freshwater component. These values are, respectively, about 14% and 5.2% of the total river outflow from Taiwan, and fall within the ranges reported elsewhere. We realize that the SGD sampling sites were not distributed evenly and that seasonal data were not obtained for most sites. However, Moosdorf *et al*.^20^ provided the only other estimate of annual fresh groundwater discharge from Taiwan at 5,486 m^3^ per m of coastline. This compares with our freshwater SGD component of 3,200 ± 4,000 m^3^/m/a. Considering the large uncertainty, the agreement is reasonable. We thus went on to look at chemical species with the understanding that an uncertainty of a factor of two is to be expected. The percentage DO saturation (DO (%)) of the SGD and local surface seawater are plotted vs salinity in Fig. 2. Again, the average DO (%), 67.6 ± 21.9, is considerably lower than that of the local surface seawater (95 ± 6.47; Table 1). In general, the DO (%) in the SGD is lower when the salinity is lower (Fig. 2a; p < 0.001) because of the consumption of DO due to decomposition of organic matter in the subterranean environment isolated from the atmosphere.

**Table 1:** Concentrations of parameters measured in the submarine groundwater and local surface seawater.

	Submarine Groundwater			Local Surface Seawater		
	range	mean ± std	n	range	mean ± std	n
S	0.008–34.8	21.92 ± 11.43	278	18.2–36.8	32.5 ± 2.42	125
DO (%)	8.3–109	67.6 ± 21.9	218	72.6–106	95 ± 6.47	106
NO_3_ (μM)	<0.02–280	27.4 ± 54.4	231	<0.02–24.4	4.84 ± 5.08	110
NO_2_ (μM)	<0.02–46.3	1.82 ± 4.95	218	<0.02–4.19	0.69 ± 0.76	109
NH_4_^+^ (μM)	0.14–3042	92.4 ± 387	131	0.35–1653	48.38 ± 205	84
PO_4_ (μM)	<0.02–33.7	0.88 ± 2.44	225	<0.05–2.70	0.55 ± 0.48	107
SiO_2_ (μM)	0.01–221	64.2 ± 58.3	224	0.37–147	10.8 ± 17.9	110
N_2_O (nM)	3.56–91	10.6 ± 14.7	51	3.64–70.9	8.7 ± 14.0	22
CH_4_(nM)	1.05–3994	523.1 ± 1231	13	3.84–1493	240 ± 554	7
DOC(μM)	24–527	114 ± 112	31	26–130	84 ± 27	9
pH	6.59–8.73	7.81 ± 0.29	166	7.27–8.40	8.10 ± 0.14	97
TA(μmol/kg)^a^	594–8579	3438 ± 1417	134	1505–4760	2343 ± 358	87
DIC(μmol/kg)	352–8675	3193 ± 1373	122	1246–4108	2040 ± 363	86
pCO_2_(μatm)	221–112455	4729 ± 13163	122	145–3732	477 ± 479	86

^a^Taken from Chen *et al*.^15^.

Decomposition of organic matter leads to the release of nutrients. As a result, the average NO_3_ concentration, 27.4 ± 54.4 μM, is much higher than that of the average local surface seawater (4.84 ± 5.08 μM; Table 1). The NO_3_ in the SGD increases when the salinity decreases (Fig. 2b; p < 0.001). In fact, the average NO_3_ concentration in the local surface seawater is higher than those generally found in waters surrounding Taiwan^21–23^ (<2 μM). Although there are other sources of NO_3_ such as riverine input and acid rain^24^, the SGD has likely played a role.

NO_2_, NH_4_ and N_2_O are all reduced forms of NO_3_ and the average NH_4_ concentration (92.4 ± 387 μM) is much higher than that of NO_3_ (27.4 ± 54.4 μM; Table 1). The presence of NO_3_ and NO_2_, however, indicates that much, but not all, of the nitrogen released as a result of organic matter decomposition has been reduced to NH_4_. Concentrations of NO_2_, NH_4_ and N_2_O are much higher in the SGD compared to those found in the local surface seawater (Table 1). Higher NO_2_ and N_2_O values are generally found when the salinity is lower (Fig. 2c, p < 0.01 for NO_2_, and Fig. 2e, p < 0.025 for N_2_O, respectively) but the pattern is not as clear in the case of NH_4_ (p < 0.25; Fig. 2d). For PO_4_ the average concentration in the SGD (0.88 ± 2.44 μM) is higher than that in the local surface seawater (0.55 ± 0.48 μM; Table 1) with waters surrounding Taiwan having the lowest value^21^ (<0.4 μM). Worth noting is that the average (NO_3_ + NO_2_ + NH_4_)/PO_4_ ratio of 136 is much higher than the Redfield ratio of 16. This is consistent with the notion that the surface waters of rivers entering the East China Sea and the South China Sea have an average N/P ratio higher than 100^25,27^. In addition, P is removed from groundwater more easily than N^27^.

Along with nitrogen and phosphorus silicate is also a major macronutrient in the oceans, and silicate concentrations in the SGD also increase with decreasing salinity (p < 0.001; Fig. 2g). The average SiO_2_ concentration in the SGD (64.2 ± 58.3 μM) is also significantly higher than the average concentration in the local surface seawater (10.8 ± 17.9 μM; Table 1). Several high values above 50 μM are found in the local surface seawater compared with the generally low value of <5 μM found in waters surrounding Taiwan^21^. This is an indication that phytoplankton uptake is not fast enough to consume the SiO_2_ released by the SGD near its source.

In the reduced environment CH_4_ is generated. We do not have sufficient CH_4_ data (n = 13) to see a clear trend relative to the salinity but the average CH_4_ concentration (523.1 ± 1,231 nM; Table 1) in the SGD is clearly higher than that in the local surface seawater (240 ± 554 nM; Table 1). Note the CH_4_ concentrations in waters around Taiwan are around only 5 nM^28^. This indicates that the SGD inputs of CH_4_ do not have sufficient time to oxidize or to be released to the atmosphere near their sources.

**Figure 3:**
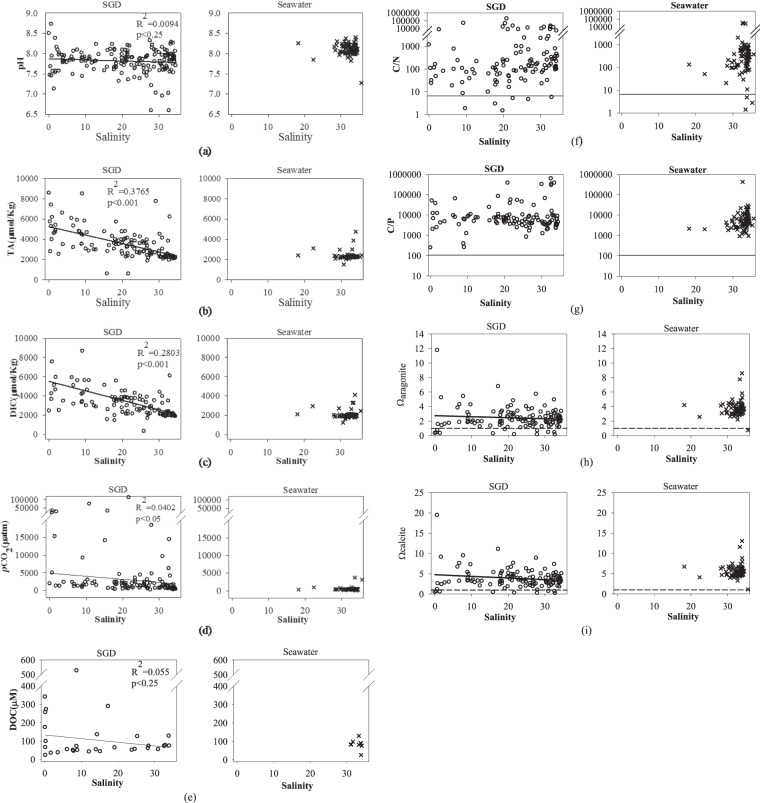
(**a**) pH, (**b**) TA, (**c**) DIC, (**d**) pCO_2_, (**e**) DOC, (f) C/N, (**g**) C/P, (**h**) Ω aragonite and (**i**) Ω calcite vs salinity in the submarine groundwater and local surface seawater, respectively.

Decomposition of organic matter generally lowers pH in the aerobic environment^29^ such as found in our case so the average pH (7.81 ± 0.29) of the SGD is slightly lower than that of the local surface seawater (8.10 ± 0.14; Table 1; Fig. 3a), which is similar to the pH of the waters surrounding Taiwan^30–33^. As for TA and DIC their values increase with the dissolution of calcareous rocks and decomposition of organic matter and it is indeed what was found. The average TA and DIC in the SGD (3,438 ± 1,417 and 3,193 ± 1,373 μmol/kg, respectively) are significantly higher than those found in the local surface seawater (2,343 ± 358 and 2,040 ± 363 μmol/kg, respectively; Table 1) and higher values are found at lower salinities (p < 0.001 in both cases; Fig. 3b,c). These TA and DIC values are more than 1,000 μmol/kg higher than those found in waters near Taiwan^33–35^.

The SGD has a high average pCO_2_ of 4,729 ± 13,163 μatm (Table 1; Fig. 3d) compared with the average of the local surface seawater (477 ± 479 μatm). The pCO_2_ shows a weak negative (p < 0.05; Fig. 3d) correlation with salinity. These values are higher than the pCO_2_ of surface waters found near Taiwan^31,33,35,36^. But, whether the surface seawater receiving the SGD is heterotrophic, i.e., whether the water is a source or sink of CO_2_ depends on the balance between carbon-consuming primary production and the excess DIC supplied by the SGD. The average C/N and C/P ratios of particulate matter in NW Pacific marginal seas are 8.8 and 152, respectively^36,37^. Based on this stoichiometry the average amount of nitrogen and phosphorus supplied by the SGD in Taiwan for each kg of water may consume 1,070 μmol/kg and 133 μmol/kg DIC, respectively. These values are much lower than the average excess DIC supported by the SGD. That is to say, primary production supported by the nutrient input from the SGD is insufficient to compensate for the high DIC and pCO_2_ supplied by the SGD. As a result, the SGD around Taiwan leads to a CO_2_ source for the atmosphere. Similar situation applies to the Jiulong and Pearl River Estuaries. Finally, decomposition of DOC also releases CO_2_. Indeed, the higher pCO_2_ values in local surface seawaters (477 ± 479 μatm; Table 1) relative to the atmosphere (~400 μatm) support this conclusion. Similar conclusion has been reported for the Pearl River Estuary^16^ and elsewhere^38,39^.

The number of DOC data is also small (n = 31) but there seems to be a trend showing high values at low salinities (p < 0.25; Fig. 3e). The average DOC in the submarine groundwater (114 ± 112 μM) is slightly higher than that in the local seawater (84 ± 27 μM; Table 1). The waters surrounding Taiwan generally have a DOC concentration below 75 μM^40,41^. Note Fig. 3e seems to indicate that the DOC is removed, hence becoming a source of nutrients and pCO_2_.

It is critical to point out that the C/N and C/P values of the SGD (Fig. 3f,g) are much higher than the Redfield Ratio. To re-iterate, the excess nutrients supplied by the SGD are insufficient to consume the excess carbon thus the SGD helps making the coastal waters heterotrophic.

Of note is that the SGD-derived DIC flux is greater than the TA flux in the Pearl River estuary, indicating that the SGD serves to reduce the CO_2_ buffering capacity of the local seawater^29^. Yet, submarine groundwaters around Taiwan the TA flux is slightly higher than the DIC flux. As a result, the SGD from Taiwan serves to increase slightly the CO_2_ buffering capacity of the local seawater. Even so, the high pCO_2_ and the high C/N and C/P ratios of Taiwan’s SGD makes it a contributor of heterotrophic nearshore waters.

**Figure 4:**
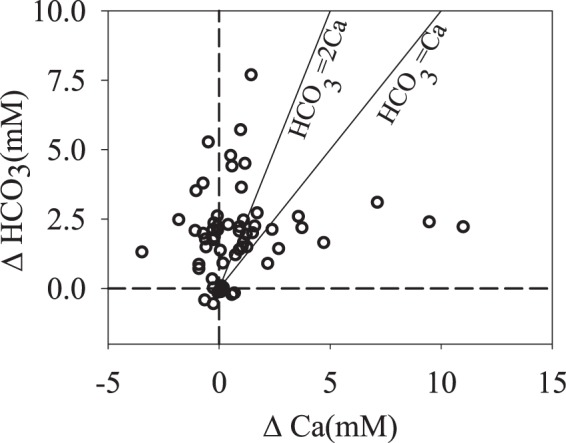
HCO_3_ plotted vs Ca for the submarine groundwater.

The percentage saturation for aragonite (Fig. 3h) and calcite (Fig. 3i) reaches a mean value of four and six, respectively for the local surface seawater but are slightly lower in the SGD. There is no doubt that the higher TA, DIC and pCO_2_ of the SGD compared to the local seawater is due to the dissolution of calcareous rocks and decomposition of organic matter in the groundwater. The decomposition of DOC must also be at play hence increasing pCO_2_. Since the submarine groundwaters do not become anoxic sulfate reduction probably has not occurred to a great extent. Figure 4 shows ΔHCO_3_ plotted vs ΔCa (local seawater is taken to be with HCO_3_ = 2.3 mM and Ca = 10.3 mM at a salinity of 35; Ca data taken from Chen *et al*.)^16^. The samples falling around the HCO_3_/Ca = 2 line reflect the dissolution of CaCO_3_. Much of the data shows an excess of HCO_3_ and Ca but the pattern is not obvious.

**Figure 5:**
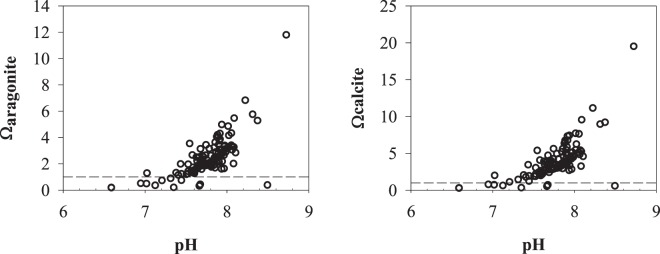
Saturation states of aragonite (Ω aragonite) and calcite (Ω calcite) vs pH for the submarine groundwater. The horizon dashed lines show the 100% saturation level.

Figure 5 shows the saturation state of aragonite and calcite plotted vs pH. Lower saturation state corresponds to lower pH, indicating that the decomposition of organic matter leads to the dissolution of calcareous rocks. The end result, however, is that the submarine groundwater is mostly highly super saturated, especially those with a pH above 7.5. The saturation state of aragonite and calcite even reach 12 and 20, respectively.

### Fluxes of nutrients and carbon

Since the properties of groundwater are not expected to show much seasonal variation as compared to the flux (e.g. Szymczycha *et al*.^39^) the above conclusions represent reasonable averages. Based on the rudimentary SGD flux value reported by Chen *et al*.^15^ the annual amount of nitrogen, phosphorus, silicate, TA and DIC export due to the SGD around Taiwan are 1.18 ± 0.83 × 10^9^, 9.3 ± 6.5 × 10^6^, 0.68 ± 0.48 × 10^9^, 3.43 ± 2.4 × 10^10^ and 3.17 ± 2.22 × 10^10^ mol, respectively (Table 2). Based on the river flow (http://gweb.wra.gov.tw/wrweb/) and the N, P data (http://wgshow.epa.gov.tw/) of the 25 largest rivers in Taiwan the total N and P fluxes are 1.12 × 10^10^ and 0.12 × 10^10^ mol/a, respectively. Simply stated, the SGD outflow is as much as 10.5% of the river outflow for N but only 0.78% for P.

**Table 2:** Annual total fluxes (mol), fluxes per m^2^ seepage area and yields (mol/km^2^ catchment area) of nutrients, TA, DIC and DOC by submarine groundwater from Taiwan, as well as Jiulong and Pearl River estuaries.

	Taiwan			Jiulong River		Pearl River		Elsewhere (Literature)
	Total Flux mol	Flux mol/m^2^	Yield mol/km^2^	Total Flux mol	Yield mol/km^2^	Total Flux mol	Yield mol/km^2^	Flux mol/m^2^ (ref.)
N (NO_3_ + NO_2_ + NH_4_)	1.18 ± 0.83 × 10^9^	0.98 ± 0.69	3.28 ± 2.3 × 10^4^	0.58–1.21 × 10^9a^ 1.83–1.95 × 10^9b^	3.90–8.23 × 10^4a^ 0.4–0.43 × 10^4b^	3.65–157 × 10^9^ (winter^d^) 0.95–40 × 10^9^ (summer^d^)	0.17–7.14 × 10^4^ (winter^d^) 0.043–1.8 × 10^4^ (summer^d^)	2.47 ± 2.16~2.63 ± 2.31 (winter, Wang *et al*.)^44^ 24.2 ± 14.9~34.2 ± 21.1 (summer, Wang *et al*.)^44^ 0–26.3 (Slomp and van Cappellen)^28^
P	9.3 ± 6.5 × 10^6^	7.75 ± 5.4 × 10^−3^	260 ± 180	2.9–6.1 × 10^5a^	20–41^a^	30–680 × 10^6^ (3) 22.6–2960 × 10^6^ (winter^d^) 5.8–767 × 10^6^ (summer^d^)	66–1500^c^ 10–1350 (winter^d^) 2.6–34.9 (summer^d^)	0.7 ± 0.6 × 10^−3^~34.7 ± 30.4 × 10^−3^ (winter, Wang *et al*.)^44^ 0–0.3 ± 0.2 × 10^−3^ (summer, Wang *et al*.)^44^ 0–0.33 (Slomp and van Cappellen)^28^
Si	0.68 ± 0.48 × 10^9^	0.57 ± 0.4	1.89 ± 1.33 × 10^4^	0.96–2.0 × 10^9a^ 2.94–3.14 × 10^9b^	6.53–13.6 × 10^4a^ 0.65–0.69 × 10^4b^	1.9–91.3 × 10^9^ (winter^d^) 0.51–23.4 × 10^9^ (summer^d^)	0.09–4.15 × 10^4^ (winter^d^) 0.02–1.06 × 10^4^ (summer^d^)	0.44 ± 0.38~0.64 ± 0.55 (winter, Wang *et al*.)^44^ 2.16 ± 1.48~3.0 ± 2.05 (summer, Wang *et al*.)^44^
TA	3.43 ± 2.4 × 10^10^	28.6 ± 20	9.5 ± 6.7 × 10^5^	0.75–1.6 × 10^10a^	5.1–10.9 × 10^5a^			46.8 (Sadat-Noori *et al*., 2016)^48^ 33–273 (Wang *et al*.)^18^ 58.8 (Stewart *et al*., 2015)^49^ 491 (Santos *et al*., 2015)^50^ 1.9–3.2 (Cyronak *et al*., 2013)^51^
DIC	3.17 ± 2.22 × 10^10^	26.4 ± 18.5	8.81 ± 6.17 × 10^5^	0.88–1.82 × 10^10a^ 2.14–2.65 × 10^10b^	6.0–12.4 × 10^5a^ 14.6–18 × 10^5b^	15.3–34.7 × 10^10c^	3.37–7.65 × 10^5c^	251 (Sadat-Noori *et al*., 2016)^48^ 44.2–327 (Wang *et al*.)^18^ 55.8 (Stewart *et al*., 2015)^49^ 394 (Porubsky *et al*., 2014)^52^ 661 (Atkins *et al*.)^39^ 91.3 (Maher *et al*., 2013)^53^ 0.6–6.9 (Cyronak *et al*., 2013)^51^ 127 (Santos *et al*., 2012)^54^ 43.8–124 (Dorsett *et al*., 2011)^55^ 730 (Moore *et al*.)^13^ 62 (Cai *et al*.)^11^
DOC	1.14 ± 1.12 × 10^9^	9.5 ± 9.3	3.17 ± 3.11 × 10^4^					0.062 ± 0.055~0.12 ± 0.11 (winter, Wang *et al*.)^44^ 0.48 ± 0.30~1.07 ± 0.66 (summer, Wang *et al*.)^44^ 197 (Sadat-Noori *et al*.)^48^ 13.1 (Stewart *et al*.)^49^ 15.3 (Santos *et al*., 2015)^50^ 23.4 (Porubsky *et al*., 2014)^52^ 8.8 (Maher *et al*., 2013)^53^ 7.7 (Santos *et al*., 2012)^54^ 6.9–9.9 (Santos *et al*.)^14^ 62 (Moore *et al*.)^13^ 18.3 (Goni and Gardner)^12^

^a^Calculated based on the flux data of Wang *et al*.^18^ and the catchment area of Jiulong River.

^b^Calculated based on the flux data of Hong *et al*.^20^ and the catchment area of Jiulong River.

^c^Obtained from Liu *et al*.^17^ and the catchment area of Pearl River.

^d^Obtained from Liu *et al*.^43^.

The Jiulong River catchment across the Taiwan Strait from Taiwan is 40.8% of Taiwan’s size. The annual SGD discharge of Jiulong River is 0.213 ± 0.058 × 10^10^ m^3^ (Wang *et al*.)^17^ which is 21.3% of the total discharge for Taiwan estimated by Chen *et al*.^15^. By way of comparison, the annual discharge of N, P, Si, TA and DIC for Jiulong River are 0.58–1.21 × 10^9^, 2.9–6.1 × 10^5^, 0.96–2.0 × 10^9^, 0.75–1.6 × 10^10^ and 0.88–1.82 × 10^10^ mol, respectively, based on the concentration and water discharge data of Wang *et al*.^17^ (Table 2). Recently Hong *et al*.^19^ also presented annual fluxes for N, Si and DIC at 1.83–1.95 × 10^9^, 2.94–3.14 × 10^9^ and 2.14–2.65 × 10^10^ mol, respectively (Table 2), comparable to the results of Wang *et al*.^17^.

Our study in Taiwan (Table 2) results in a SGD discharge of about 1.18 ± 0.83 × 10^9^ mol/a N (NO_3_ + NO_2_ + NH_4_), which is equivalent to a yield of 3.28 ± 2.3 × 10^4^ mol/a N per square kilometer of the total catchment area. This value is similar to the annual yield of Jiulong River at 3.90–8.23 × 10^4^ mol/km^2^/a calculated based on the size of the catchment area and the flux of N reported by Wang *et al*.^17^. The flux of P resulted from this study for Taiwan is 9.3 ± 6.5 × 10^6^ mol/a, and the yield is 260 ± 180 mol/km^2^/a. The P flux and yield for Jiulong River at 2.9–6.1 × 10^5^ and 20–41 mol/km^2/a^ (Table 2), respectively, are surprisingly low. As reported above, the N/P ratio obtained from this study for the submarine groundwater is 136. The flux data of Wang *et al*.^17^ for Jiulong River translate to a N/P ratio of 2000 which is extremely high although we realize that P is removed from groundwater. Our work for the Jiulong River (Table 3) results in an N (n = 11) to P (n = 9) ratio of 51 in the river basin and an N (n = 7) to P (n = 7) ratio of 27 in the estuary. Of note is that the average groundwater N and P concentrations calculated from the data of Wang *et al*.^17^ are 495 and 22.75 μM, respectively. The resulting N/P ratio is only 21.8, similar to what we found in the Jiulong River estuary but way below the reported ratio of 2,000 for the SGD by these authors.

**Table 3:** Concentrations of NO_3_, NO_2_, NH_4_ and PO_4_ for riverine (S < 2) and estuarine water (S ≧ 2) of Jiulong River.

	S < 2			S ≧ 2		
	range	mean ± std	n	range	mean ± std	n
NO_3_ (μM)	71~173	95 ± 28	11	14~50	23 ± 12	7
NO_2_ (μM)	3.0~39	14 ± 13	11	1.0~4.0	2.2 ± 0.9	8
NH_4_^+^ (μM)	11~211	55 ± 63	11	14~59	23 ± 18	6
PO_4_ (μM)	1.3~11.0	3.2 ± 3.2	9	0.5~7.6	1.8 ± 2.4	7

Liu *et al*.^16^ reported the total annual flux of P for the Pearl River at 30–680 × 10^6^ mol and Liu *et al*.^42^ obtained similar values. Liu *et al*.^42^ also reported the N fluxes for the Pearl River at 3.65–157 × 10^9^ and 0.95–40 × 10^9^ mol in summer and winter, respectively. They reported the Si fluxes at 1.9–91.3 × 10^9^ and 0.51–23.4 × 10^9^ mol in summer and winter, respectively (Table 2). The Pearl River (Fig. 1) has a large catchment area of 453,700 km^2^ which is located at the same latitude as southern Taiwan. The P flux of Liu *et al*.^16^ translates to a yield of 66–1,500 mol/km^2^/a and the results of Liu *et al*.^42^ are similar (Table 2). These results are comparable to those from Taiwan but much higher than those from the Jiulong River. The annual DIC flux of Liu *et al*.^16^ for the Pearl River is 15.3–34.7 × 10^10^ mol and the yield is 3.37–7.65 × 10^5^ mol/km^2^ which is comparable with our result in Taiwan. The reported SGD N and P in the literature are also given in Table 2, and the ranges are high. Our fluxes per m^2^ for N and P in Taiwan are at the low end of these reported values.

The total flux of Si for Taiwan is 0.68 ± 0.48 × 10^9^ mol/a compared with the larger flux of 0.96–2.0 × 10^9^ mol/a (Wang *et al*.)^17^ or 2.94–3.14 × 10^9^ mol/a (Hong *et al*.)^19^ reported for Jiulong River. As for the yield the value of 6.53–13.6 × 10^4^ mol/km^2^/a calculated based on the flux data of Wang *et al*.^17^, and the value of 0.65–0.69 × 10^4^ mol/km^2^a based on the data of Hong *et al*.^19^ bracket the yield of Taiwan at 1.89 ± 1.33 × 10^4^ mol/km^2^/a. Liu *et al*.^42^ reported the total flux and yield of Si for the Pearl River. Although their total fluxes are high their yields also bracket our results for Taiwan. The Si fluxes reported for a subtropical bay in south China^43^ are slightly higher than those for Taiwan (Table 2).

The TA and DIC fluxes for Taiwan are 3.43 ± 2.4 × 10^10^ and 3.17 ± 2.22 × 10^10^ mol/a, respectively. These values compare with 0.75–1.6 × 10^10^ and 0.88–1.82 × 10^10^ mol/a, respectively, for Jiulong Rvier based on the data of Wang *et al*.^17^ (Table 2). The yield of TA for Taiwan at 9.5 ± 6.7 × 10^5^ mol/km^2^/a is slightly higher than those for Jiulong River, at 5.1–10.9 × 10^5^ mol/km^2^/a. The reported TA fluxes elsewhere bracket our results (Table 2). The yield of DIC for Taiwan is 8.81 ± 6.17 × 10^5^ mol/km^2^/a which falls between the slightly lower value of 6–12.4 × 10^5^ mol/km^2^/a (Wang *et al*.)^17^ and the slightly higher value of 14.6–18 × 10^5^ (Hong *et al*.)^19^ for the Jiulong River. The Pearl River basin also has an abundance of calcareous rocks. The DIC yield (3.37–7.65 × 10^5^ mol/km^2^/a), nevertheless, is smaller compared to our result in Taiwan. This is perhaps because the weathering is weaker in the less steep Pearl River basin. This points to the difficulty of comparing the total flux or yield. It is yet not possible to compare data per unit area of the ocean floor or per unit length of the coastal line. In terms of flux per m^2^ of the seepage area, however, our DIC flux falls in the range reported elsewhere as shown in Table 2. Our DOC flux (9.5 ± 9.3 mol/m^2^/a) is also comparable with those reported in the literature (Table 2).

## Conclusions

The concentrations, fluxes and yields of N, P, Si, TA and DIC for the SGD in Taiwan have been reported for the first time, and these values are broadly comparable with the data in the literature. The nutrients supplied by the SGD are insufficient to compensate the DIC supported at the same time. As a result, the SGD around Taiwan leads to a source of CO_2_ for the atmosphere in the coastal seas. Similar situation exists in the Jiulong and Pearl River estuaries in Southeast China, and perhaps in other coastal regions around the world as well.

## Methods

Geologically Taiwan is relatively young. The collision of the Philippine Arc and the Asian continent gave rise to the Central Range of Taiwan, and the orogenesis is still going^44^. The population is 23 million. The western part of Taiwan is mainly covered by undeformed sediments, and is heavily populated. Less populated is Southern Taiwan where the coasts are largely covered by coral reefs. Eastern Taiwan has a coastal range, and the less populated coasts are mainly rocky.

Preliminary sampling of the SGD in Taiwan was performed from 2004 to 2016. Twenty sampling sites around the coastal areas Taiwan are shown in Fig. 1. Measurements of SGD fluxes were reported in Chen *et al*.^15^. Submarine groundwater samples for chemical analysis were drawn by a device designed by Zhang and Satake^45^ mostly on the sandy coast. At one site 350 m off SW Taiwan divers collected freshwater (S = 0.008) at a water depth of 8 m. The corresponding local surface seawater sample was also collected. Samples for NO_3_, NO_2_, NH_4_ and PO_4_ were collected in Jiulong River and its estuary in 2008. Preserved samples, with saturated HgCl_2_ added, were brought back and measured in the laboratory with details given in Chen^21^, Yang *et al*.^41^ and Tseng *et al*.^46^ and 2017^28^. The percentage DO saturation (DO (%)) was calculated based on the solubility equation of Chen^47^. The HCO^−^_3_ and pCO_2_ were calculated based on pH and TA using the CO2SYS program.

## References

[1] Burnett WC, Taniguchi M, Oberdorfer J (2001). Measurement and significance of the direct discharge of groundwater into the coastal zone. J Sea Res.

[2] Burnett WC, Bokuniewicz H, Huettel M, Moore WS, Taniguchi M (2003). Groundwater and pore water inputs to the coastal zone. Biogeochemistry.

[3] Church TM (1996). An underground route for the water cycle. Nature.

[4] Gu HQ, Moore WS, Zhang L, Du JZ, Zhang J (2012). Using radium isotopes to estimate the residence time and the contribution of submarine groundwater discharge (SGD) in the Changjiang effluent plume, East China Sea. Cont Shelf Res.

[5] Rodellas V, Garcia-Orellana J, Masque P, Feldman M, Weinstein Y (2015). Submarine groundwater discharge as a major source of nutrients to the Mediterranean Sea. P Natl Acad Sci USA.

[6] Taniguchi M, Burnett WC, Cable JE, Turner JV (2002). Investigation of submarine groundwater discharge. Hydrol Process.

[7] Wang XL (2014). An estimation of nutrient fluxes via submarine groundwater discharge into the Sanggou Bay-A typical multi-species culture ecosystem in China. Mar Chem.

[8] Zhang J, Satake H (2002). Submarine groundwater seepage in Toyama. Aquabiology.

[9] Zhang L (2013). Pore water nutrient characteristics and the fluxes across the sediment in the Pearl River estuary and adjacent waters, China. Estuar Coast Shelf S.

[10] Moore WS (2010). The effect of submarine groundwater discharge on the ocean. Annu Rev Mar Sci.

[11] Cai WJ, Wang YC, Krest J, Moore WS (2003). The geochemistry of dissolved inorganic carbon in a surficial groundwater aquifer in North Inlet, South Carolina, and the carbon fluxes to the coastal ocean. Geochim Cosmochim Ac.

[12] Goni MA, Gardner LR (2003). Seasonal dynamics in dissolved organic carbon concentrations in a coastal water-table aquifer at the forest-marsh interface. Aquat Geochem.

[13] Moore, W. S., Blanton, J. O. & Joye, S. B. Estimates of flushing times, submarine groundwater discharge, and nutrient fluxes to Okatee Estuary, South Carolina. *J Geophys Res-Oceans* **111**, 10.1029/2005JC003041 (2006).

[14] Santos IR, Burnett WC, Dittmar T, Suryaputra IGNA, Chanton J (2009). Tidal pumping drives nutrient and dissolved organic matter dynamics in a Gulf of Mexico subterranean estuary. Geochim Cosmochim Ac.

[15] Chen CTA (2018). Submarine groundwater discharge around Taiwan. Acta Oceanlologica Sinica.

[16] Liu Q (2012). How significant is submarine groundwater discharge and its associated dissolved inorganic carbon in a river-dominated shelf system?. Biogeosciences.

[17] Wang GZ (2015). Net subterranean estuarine export fluxes of dissolved inorganic C, N, P, Si, and total alkalinity into the Jiulong River estuary, China. Geochim Cosmochim Ac.

[18] Cai PH (2015). Using Ra-224/Th-228 disequilibrium to quantify benthic fluxes of dissolved inorganic carbon and nutrients into the Pearl River Estuary. Geochim Cosmochim Ac.

[19] Hong QQ, Cai PH, Shi XM, Li Q, Wang GZ (2017). Solute transport into the Jiulong River estuary via pore water exchange and submarine groundwater discharge: New insights from Ra-224/Th-228 disequilibrium. Geochim Cosmochim Ac.

[20] Moosdorf N, Stieglitz T, Waska H, Durr HH, Hartmann J (2015). Submarine groundwater discharge from tropical islands: a review. Grundwasser.

[21] Chen CTA (2008). Distributions of nutrients in the East China Sea and the South China Sea connection. J Oceanogr.

[22] Chen CTA, Wang SL (2006). A salinity front in the southern East China Sea separating the Chinese coastal and Taiwan Strait waters from Kuroshio waters. Cont Shelf Res.

[23] Naik H, Chen CTA (2008). Biogeochemical cycling in the Taiwan Strait. Estuar Coast Shelf S.

[24] Chen CTA, Wang BJ, Hsu HC, Hung JJ (1994). Rain and lake waters in Taiwan: Composition and Acidity. Terrestrial, Atmospheric and Oceanic Sciences.

[25] Chen CTA, Wang SL (1999). Carbon, alkalinity and nutrient budgets on the East China Sea continental shelf. J Geophys Res-Oceans.

[26] Chen CTA, Wang SL, Wang BJ, Pai SC (2001). Nutrient budgets for the South China Sea basin. Mar Chem.

[27] Slomp CP, Van Cappellen P (2004). Nutrient inputs to the coastal ocean through submarine groundwater discharge: controls and potential impact. J Hydrol.

[28] Tseng HC, Chen CTA, Borges AV, DelValls TA, Chang YC (2017). Methane in the South China Sea and the Western Philippine Sea. Cont Shelf Res.

[29] Liu Q (2017). Carbonate system biogeochemistry in a subterranean estuary - Waquoit Bay, USA. Geochim Cosmochim Ac.

[30] Chen CTA, Yeh YT, Chen YC, Huang TH (2015). Seasonal and ENSO-related interannual variability of subsurface fronts separating West Philippine Sea waters from South China Sea waters near the Luzon Strait. Deep-Sea Res Pt I.

[31] Hong HS (2011). Source water of two-pronged northward flow in the southern Taiwan Strait in summer. J Oceanogr.

[32] Huang TH, Chen CTA, Zhang WZ, Zhuang XF (2015). Varying intensity of Kuroshio intrusion into Southeast Taiwan Strait during ENSO events. Cont Shelf Res.

[33] Sheu DD (2009). Riding over the Kuroshio from the South to the East China Sea: Mixing and transport of DIC. Geophys Res Lett.

[34] Bai Y (2015). Intrusion of the Pearl River plume into the main channel of the Taiwan Strait in summer. J Sea Res.

[35] Chou, W. C. *et al*. Transport of the South China Sea subsurface water outflow and its influence on carbon chemistry of Kuroshio waters off southeastern Taiwan. *J Geophys Res-Oceans* **112** (2007).

[36] Pan YW, Fan W, Huang TH, Wang SL, Chen CTA (2015). Evaluation of the sinks and sources of atmospheric CO2 by artificial upwelling. Sci Total Environ.

[37] Chen CTA, Lin CM, Huang BT, Chang LF (1996). Stoichiometry of carbon, hydrogen, nitrogen, sulfur and oxygen in the particulate matter of the western North Pacific marginal seas. Mar Chem.

[38] Atkins ML, Santos IR, Ruiz-Halpern S, Maher DT (2013). Carbon dioxide dynamics driven by groundwater discharge in a coastal floodplain creek. J Hydrol.

[39] Szymczycha B, Maciejewska A, Winogradow A, Pempkowiak J (2014). Could submarine groundwater discharge be a significant carbon source to the southern Baltic Sea?. Oceanologia.

[40] Liu Q (2014). Estimating dissolved organic carbon inventories in the East China Sea using remote-sensing data. J Geophys Res-Oceans.

[41] Yang LY, Chen CTA, Lui HK, Zhuang WE, Wang BJ (2016). Effects of microbial transformation on dissolved organic matter in the east Taiwan Strait and implications for carbon and nutrient cycling. Estuar Coast Shelf S.

[42] Liu JA, Du JZ, Wu Y, Liu SM (2018). Nutrient input through submarine groundwater discharge in two major Chinese estuaries: the Pearl River Estuary and the Changjiang River Estuary. Estuar Coast Shelf S.

[43] Wang G, Han A, Chen L, Tan E, Lin H (2018). Fluxes of dissolved organic carbon and nutrients via submarine groundwater discharge into subtropical Sansha Bay, China. Estuarine, Coastal and Shelf Science.

[44] Hsü, K. J. & Chen, H. H. *Geologic Atlas of China: An Application of the Tectonic Facies Concept to the Geology of China*. 262 and 23 Atlas Sheets (Elsevier, 1999).

[45] Zhang, J. & Satake, H. In Land and Marine Hydrogeology (eds Taniguchi, M., Wang, k. & Gamo, T.) 45–60 (ELSEVIER B.V., 2003).

[46] Tseng HC (2016). Distributions and sea-to-air fluxes of nitrous oxide in the South China Sea and the West Philippines Sea. Deep-Sea Res Pt I.

[47] Chen, C. T. In Solubility Data Series Vol. 7 (ed. Battino, R.) 41–55 (Pergamon Press, 1981).

